# Utility of teleconsultation in accessing eye care in a developing country during COVID-19 pandemic

**DOI:** 10.1371/journal.pone.0245343

**Published:** 2021-01-14

**Authors:** Hassan Mansoor, Saad Alam Khan, Tayyab Afghani, Muhammad Zaman Assir, Mahmood Ali, Wajid Ali Khan

**Affiliations:** 1 Al-Shifa Trust Eye Hospital, Rawalpindi, Pakistan; 2 Department of Medicine, Allama Iqbal Medical College, University of Health Sciences, Lahore, Pakistan; Singapore Eye Research Institute, SINGAPORE

## Abstract

**Objective:**

To evaluate the utility of teleconsultation in the provision of eye care services during the COVID-19 lockdown. Disparities in the consultation burden of sub-specialities and socio-demographic differences in teleconsultation utilization were also assessed.

**Methods:**

Al-Shifa Trust Eye Hospital Rawalpindi began audio and video teleconsultation using broadband telecommunication services during the lockdown. Patients’ and consultations’ data gathered during the first three weeks after the commencement of this programme were compared with data from the four weeks prior to lockdown. The weekly consultation ratio and overall consultation burden of sub-specialities were measured. Chi-Square tests of association determined the relationship between different variables (socioeconomic status and consultation characteristics) and consultation modality (on-site vs online).

**Results:**

In total, 17507 on-site consultations (4377/week) were conducted compared to 1431 teleconsultations (477/week), which maintained 10.89% of the weekly pre-lockdown eye care services. The post-lockdown teleconsultation programme saw a relatively higher percentage of service utility among female (47.09% vs 44.71%), younger-age (31.33±19.45 vs 41.25±23.32 years) and higher-socioeconomic-status (32.21% vs 0.30%) patients compared to pre-lockdown on-site consultations. The most common indication for teleconsultation was red-eye (16.70%). While cornea and glaucoma clinics maintained most of the pre-lockdown services (30.42% and 29% respectively), the highest dropout was seen in optometric and vitreoretinal services supporting only 5.54% and 8.28% of pre-lockdown services, respectively.

**Conclusion:**

Digital initiatives could partially maintain eye care services during the lockdown. Focused strategies to improve teleconsultation utilization are required during the pandemic and beyond.

## Introduction

With the ever-maturing development of several digital technologies that address clinical problems and diseases, 2020 should have been the harbinger of an exciting decade in medicine and science. These digital technological advancements included big-data analytics [1], blockchain technology [2] and the Internet of Things (IoT) with next-generation telecommunication networks (e.g., 5G) [3]. However, the world now confronts a monumental health crisis in the form of the outbreak of a novel corona virus-induced infectious respiratory disease (COVID-19) [4]. Amid the COVID-19 pandemic, non-essential and non-critical healthcare services have seen a forced shutdown in most high-risk countries to limit the spread of the disease [5]. However, such drastic yet inevitable measures may not be sustained indefinitely. Hence countries around the world are trying to adapt to technology-driven medical consultation and treatment approaches to counter the pandemic’s impact [6, 7].

The use of telecommunication technology to provide clinical services remotely is known as telemedicine [8–10]. Telemedicine allows the application of clinical science by telephone, the internet, or other networks [8, 11]; it has been used in different medical specialities for the diagnosis and treatment of diseases, as well as research, and education [12–15]. Telemedicine is gaining popularity in the COVID-19 era [16–24]. Before the current pandemic, 74% of patients did not know that they could avail the telemedicine option in their physicians' practices [25]. However, this scenario has changed with social distancing measures, as some leading telehealth platforms have reported an increase between 257% and 700% in the number of virtual patient visits, which may correlate with the geographical impact of COVID-19 [25].

Telemedicine also provides an opportunity for ophthalmologists to conduct consultations in far-flung and underserved areas that may not otherwise have access to specialized eye care [8, 12, 26]. Teleophthalmology services can be provided by the store-and-forward method or in real-time [26–28]. In store-and-forward mode, patients’ electronic medical records, laboratory results, slit lamp and fundus images, and audio or video clips (e.g., eye movements, pupillary examination) are forwarded to a specialist who reviews the referral at a convenient time [26]. In comparison, real-time teleophthalmology adopts interactive services, such as audio telephone calls or videoconferencing and remote monitoring methods [25, 26]. On the other hand, a hybrid technique combines both store-and-forward and real-time teleexamination to increase the efficiency of teleophthalmology services [26, 27]. Using these techniques, teleophthalmology has been used for screening and diagnosing diabetic retinopathy, age-related macular degeneration and retinopathy of prematurity; cataract and glaucoma screening; anterior segment imaging; telementoring and low vision consultation [8, 9, 26, 29]. Even during the COVID-19 pandemic, teleophthalmology has been deployed for triage, consultation and reassurance [30–34]; visual acuity assessment [35]; and contact lens services [36]. As ophthalmologists have an increased risk of becoming infected with coronavirus due to their close contact with the patients during a slit-lamp examination, they must adapt to teleconsultations to combat COVID-19.

Currently, smartphones have become an integral part of our daily life [9]. A high mobile-broadband penetration rate has also been reported worldwide [9, 37]. With a teledensity of over 78% and 165 million cellular subscribers in Pakistan [38], mobile connectivity and economical broadband services present a unique opportunity for the continued provision of eye care through audio and video telephone calls, at a time when the country is under lockdown. This real-time teleconsultation will help doctors and hospital administrations achieve some important objectives, such as triage of acute problems for referrals, reassurance, reevaluation of care plans, as well as rescheduling of upcoming appointments or surgical procedures [25, 39]. Since a virtual consultation avoids physical crowding of hospitals, it may also limit the spread of the highly transmissible coronavirus and ensure the safety of health care professionals as well as the patients [40].

During the COVID-19 pandemic, some individuals could be more vulnerable to severe disease and might take a much longer road to recovery due to their limited access to healthcare facilities. Health equity is the pledge to lessen disparities in health outcomes and their determinants, including sociodemographic aspects [41–43]. While government actions such as economic relief packages and social uplift programmes, are essential to deal with the emerging COVID-19 economic and social crisis, it is imperative to harness the potential of digital technology to identify factors that could promote health equity. Reducing health inequities would help achieve better healthcare for everyone during the pandemic.

This study is aimed to estimate the magnitude and determinants of the audio and video teleconsultations’ utility for the provision of eye care services in Pakistan during the COVID-19 pandemic. We studied the sociodemographic characteristics of the patients and utilization trends of the sub-speciality services before and during the lockdown in order to identify factors that may hamper eye care equity and access during the COVID-19 pandemic. These factors can then be targeted with focused strategies to expand and ensure the equitable utility of teleconsultation services.

## Materials and methods

### Study setting

Pakistan is a minimally resourced, low middle-income country situated in South Asia [44]. With an overall area of 796,096 km^2^, it is divided into four provinces (Punjab, Sindh, Balochistan and Khyber Pakhtunkhwa) [45]. Amongst other eye care facilities in Pakistan, Al-Shifa Trust is a non-political, non-governmental, and not-for-profit organization involved in the delivery of high-quality eye care services since 1991. It has four tertiary eye care hospitals that are situated in strategic locations (Rawalpindi, Sukkur, Kohat and Muzaffarabad) to cover the eye care needs of the communities throughout the country. Routinely, Al-Shifa Trust Eye Hospitals provide generalized and sub-speciality eye care services to all the socioeconomic segments of the society. These services include outdoor, indoor and emergency departments along with the sub-speciality clinics for ocular problems related to the Cornea and Refractive Surgery, Vitreoretinal diseases, Glaucoma, Paediatric Ophthalmology, Oculoplastics and Orbital Surgery.

### Teleconsultation

**Fig 1:**
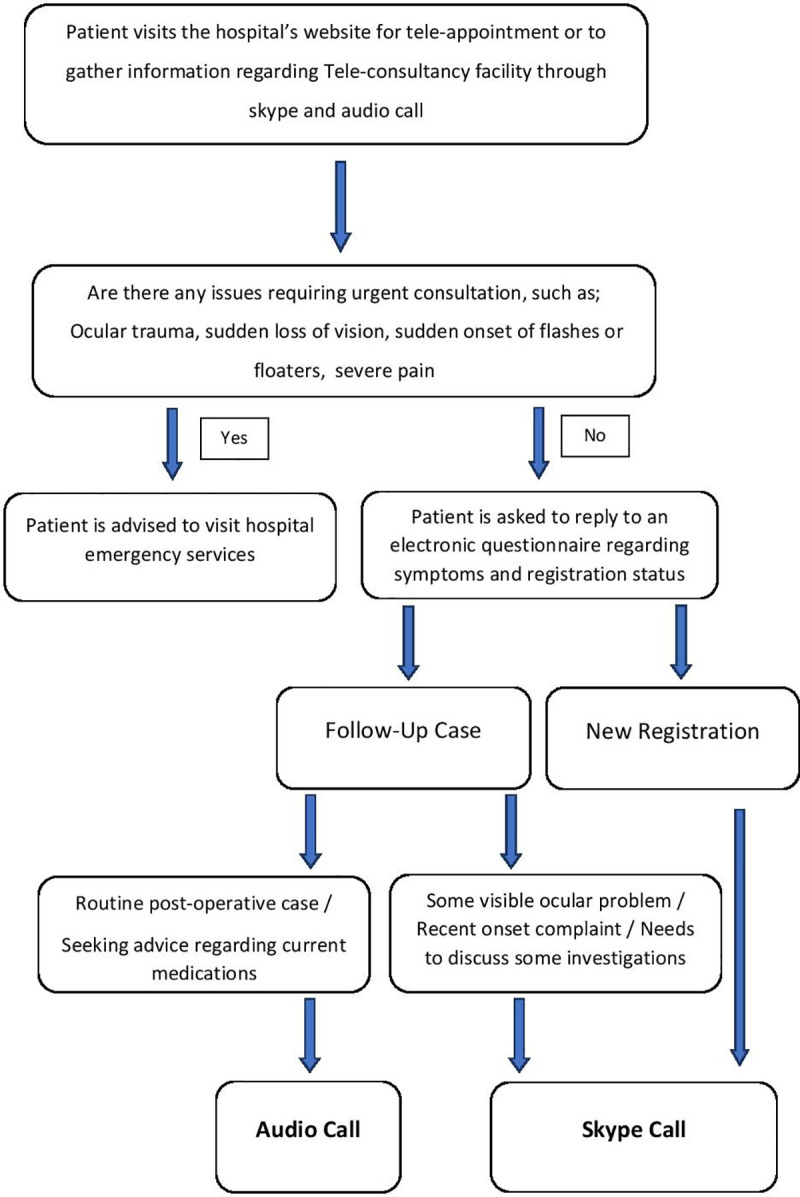
An overview of the teleconsultation programme.

Since the COVID-19 pandemic in Pakistan led to a forced shutdown of all non-essential and non-critical healthcare services on 23^rd^ March 2020, the Al-Shifa Trust Eye Hospital Rawalpindi started an online audio and video teleconsultation programme on 24^th^ March 2020 to maintain eye care services. Using mobile connectivity and broadband services, the virtual teleconsultation programme connected patients with the consultants through social networking applications, such as Skype, for video assessment and patient-doctor interactions as well as by audio phone-calls. Eight consultants of different sub-specialities were designated an hour every day six days a week (Monday to Saturday) to provide audio and video teleconsultation. The Skype ID, consultants’ schedules and teleconsultation information, such as booking procedure and contact details were advertised through the social networking platforms, such as Facebook and Instagram, as well as through short message service (SMS). One thousand SMS were sent out every day after the lockdown to random patients from the hospital’s data repository. The patients could also visit the hospital’s website (www.alshifaeye.org) to gather information regarding the teleconsultation programme or book a tele-appointment. On the website, the patients were asked to fill in an electronic questionnaire regarding symptoms and registration status (new or follow-up). These teleconsultations also provided prescriptions and ensured emergency referrals, if deemed necessary (Fig 1).

### Study objectives

The utility of teleconsultation during the lockdown was determined by the comparative assessment of a primary outcome variable, i.e, the consultation burden, in normal circumstances (before lockdown), and during the lockdown.

The consultation burden was evaluated based on the weekly consultation ratio (WCR), which was the total number of general and sub-speciality consultations during the study duration/total study duration (weeks). The relative decrease in the sub-speciality WCR was measured by calculating the change in WCR as a proportion of the total WCR for the pre-lockdown study duration. The mathematical formula for calculating percentage decline in sub-speciality specific WCR is given below:

$$\frac{wcr(pre)-wcr(post)}{wcr(pre)}x\;100$$

where wcr(pre) is sub-speciality WCR before lockdown and wcr(post) is the corresponding sub-speciality WCR after lockdown.

### Consultation burden before the lockdown

A retrospective analysis of Al-Shifa Trust Eye Hospital Rawalpindi’s record from 24^th^ February 2020 to 22^nd^ March 2020 (4 weeks) was carried out to calculate the consultation burden classified for different sub-speciality departments. Age and gender distribution, registration day, registration status (new or follow-up), patients’ socioeconomic status (the hospital categorized it based on the annual income of the patient as either low income/free treatment, middle income/subsidized payment or high income/private patients) as well as the consultation indication of the patients during the study period were reviewed.

### Consultation burden during the lockdown

After the commencement of the teleconsultation programme, information related to abovementioned study parameters was prospectively gathered for three weeks. Subsequently, the collected data were analysed to calculate the consultation burden. During the teleconsultation, the patients were also asked about how they came to know about the programme. The number of patients requiring an urgent referral during the teleconsultation programme was also determined.

### Cost-effectiveness

A cost analysis was conducted to determine the weekly expenses (48 hours/week) of the hospital for both consultation modalities (on-site vs teleconsultation). The details, such as hourly consultation charges/consultant, internet expenses, utility and publicity expenses, equipment maintenance and repair charges, and equipment and building depreciation charges, for both components of the study duration were obtained from the audit department of the hospital in the form of financial records. The costs, which were equivalent for both consultation modalities, were excluded from the final analysis. Weekly expenses were calculated on an hourly basis and obtained after dividing the weekly expenses of every category by the total number of working hours/week (48 hours). Moreover, the hourly consultancy charges/consultant for 1 week were calculated by dividing the total hourly salaries of the participating consultants (on-site vs teleconsultation) in 1 week by the number of consultants for each consultation modality.

### Statistical analysis

All analyses were carried out in Statistical Package for Social Sciences (SPSS) version 17. For descriptive analysis, the frequencies and percentages were used for categorical variables. The arithmetic mean and standard deviation with ranges described the quantitative variables. A Chi-square test with a 95% confidence interval was applied for the inferential analysis to show relationships and trends regarding the modality of consultation (online vs on-site) in categorical variables. On the other hand, an independent samples Student’s t-test was applied to the quantitative variable to determine its association with the modality of consultation (online vs on-site) at a 95% confidence interval.

## Results

In total, 17507 on-site consultations were conducted at Al-Shifa Trust Eye Hospital Rawalpindi during the four weeks prior to lockdown compared to 1431 teleconsultations that were conducted in three weeks following the commencement of lockdown. Hence, the teleconsultation programme maintained 10.89% of the pre-lockdown weekly eye care services. The predominant mode of teleconsultation was through video calls (n = 1279, 89.37%), whereas the remainder were audio calls.

### Sociodemographic characteristics of patients

The predominant gender during the pre and post-lockdown study duration was male, although an increase in females from 44.71% pre-lockdown to 47.09% post-lockdown was observed (Table 1).

**Table 1:** Sociodemographic and consultation characteristics of patients.

Variables	Pre-Lockdown duration % (n)	Post-Lockdown duration % (n)
	n = 17507	n = 1431
**Gender**		
Male	55.29 (9681)	52.91 (757)
Female	44.71 (7826)	47.09 (674)
**Socioeconomic Status**		
Free Treatment/Low Income	75.19 (13165)	33.12 (474)
Subsidized Payment/Middle Income	20.19 (3536)	26.90 (385)
Private Patients/High Income	0.30 (53)	32.21 (461)
Others^a^	4.32 (753)	7.77 (111)
**Registration Status**		
New	46.19 (8088)	45.07 (645)
Follow-Up	53.81 (9419)	54.93 (786)
**Consultation Day**		
Monday	21.96 (3845)	16.41 (235)
Tuesday	19.78 (3463)	15.42 (221)
Wednesday	15.21 (2663)	18.91 (271)
Thursday	15.23 (2666)	15.79 (225)
**Weekdays (Monday to Thursday)**	72.18 (12637)	66.53 (952)
Friday	12.40 (2171)	15.93 (228)
Saturday	15.42 (2699)	17.54 (251)
**Weekend (Friday and Saturday)**	27.82 (4870)	33.47 (479)

^a^ Others include Panel medical services and Private insurance companies.

An age-wise comparison of the study participants revealed that the participants of the post-lockdown teleconsultation programme were younger than the pre-lockdown participants (p<0.001), with a mean age of 31.33±19.45 years (5–68 years) and 41.25±23.32 years (1–100 years), respectively (Table 2).

**Table 2:** Teleconsultation associations.

Variable	Pre Lockdown % (N)	Post-Lockdown % (N)	χ^2^(df)	p-value	Effect size
**Age** ^**a**^	41.25 (23.32)	31.33 (19.45)	15.65 (18936)	<0.001*	
**Gender**					
Male	55.30 (9688)	52.90 (757)	3.18 (1)	0.08	-
Female	44.70 (7819)	47.10 (674)			
**Socioeconomic Status**					
Free treatment/ Low income	75.20% (13162)	33.10 (474)	5408.99 (3)	<0.001*	0.53
Subsidized payment/ Middle income	20.20 (3540)	26.90 (385)			
Private patients/ High income	0.30 (48)	32.20 (461)			
Others	4.30 (757)	7.80 (111)			
**Consultation Department**					
General Ophthalmology	40.00 (7003)	29.63 (424)	59.63 (1)	<0.001*	-0.06
Sub-speciality	60.00 (10504)	70.37 (1007)			
**Registration Status**					
New	46.20 (8090)	45.10 (645)	0.69 (1)	0.41	-
Follow-Up	53.80 (9417)	54.90 (786)			

*Significant results

^a^ Mean (SD) were reported for age, and independent samples student t-test was applied

While more than thirteen thousand patients (n = 13165, 75.19%) were registered in the “Free Treatment” hospital category during the pre-lockdown study, the teleconsultation programme saw a total of 474 (33.12%) consultations in the same category. Conversely, the number of private patients increased from 0.30% (n = 53) of pre-lockdown duration to 32.21% (n = 461) of post-lockdown study duration (p<0.001), which accounted for an ~9-fold increase in the absolute number of private patients (n) utilizing the teleconsultation service (Tables 1 and 2).

### Consultation burden

Most consultations during the pre-lockdown study duration and in the teleconsultation programme were follow-up based (n = 9419, 53.81% and n = 786, 54.93%, respectively) and were conducted during weekdays (Monday to Thursday) (n = 12637, 72.18% and n = 952, 66.53%, respectively) (Table 1). There were 4377 consultations per week (weekly consultation ratio) during the pre-lockdown study duration compared to 477 consultations per week in the teleconsultation programme.

### Indications for consultation

The most common indication for consultation during the pre-lockdown study was refractive error (n = 4937, 28.20%) followed by cataract (n = 3344, 19.10%) while it was red-eye (n = 239, 16.70%) for the post-lockdown study. More than 150 patients (n = 154, 10.76%) were referred to the Emergency Department of the hospital facility during the teleconsultation program while 13.77% patients (n = 197) were advised a deferred evaluation once the lockdown restrictions were relaxed. The details of the consultation indications are given in Table 3.

**Table 3:** Consultation indications.

Consultation Indications	Pre-Lockdown % (n)	Post-Lockdown % (n)	Emergency Referral % (n)^a^	Deferred Evaluation % (n)
	n = 17507	n = 1431	n = 154	n = 197
Refractive error	28.20 (4937)	12.99 (186)	-	41.40 (77)
Red-Eye	12.82 (2241)	16.70 (239)	14.64 (35)	6.54 (15)
Cataract	19.10 (3344)	14.46 (207)	3.84 (8)	18.24 (44)
Stable Glaucoma (follow-up)	6.99 (1225)	15.23 (218)	4.58 (10)	14.63 (32)
Diabetic Retinopathy	11.80 (2066)	12.08 (173)	7.51 (13)	9.65 (17)
Trauma	4.70 (823)	9.36 (134)	32.08 (43)	3.32 (4)
Infections	3.10 (543)	4.33 (62)	23.9 (15)	3.16 (2)
Others^b^	13.29 (2328)	14.81 (212)	14.15 (30)	3.06 (6)

^a^ Emergency referral during an online consultation

^b^ Others include sudden vision loss, severe ocular pain, suspected metamorphopsia, suspected glaucoma, and follow up patients for eye surgeries.

### Sub-speciality distribution

**Fig 2:**
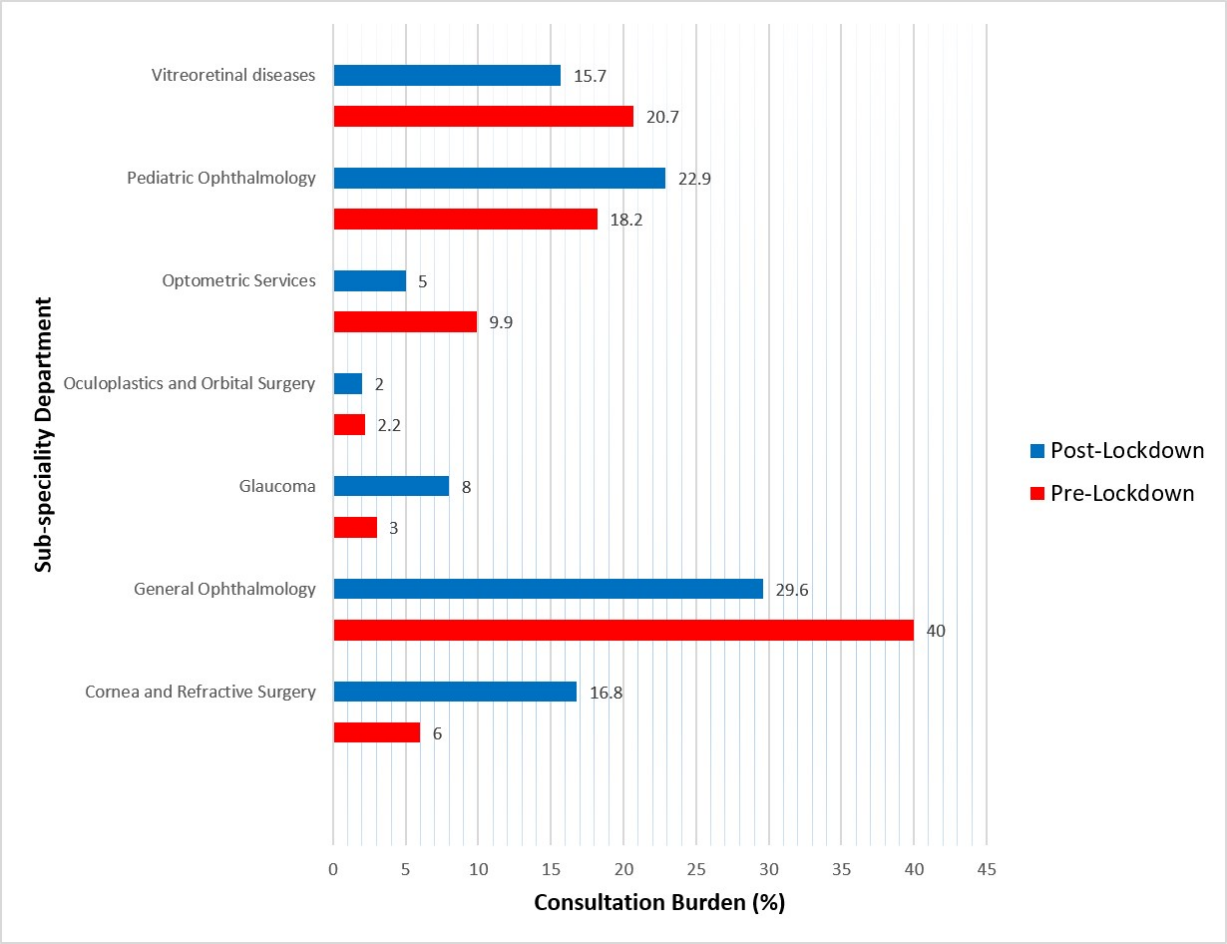
Sub-speciality distribution of consultation burden (%).

The General Ophthalmology department carried out the majority of the consultations pre-lockdown (n = 7004, 40.00%) and post-lockdown (n = 423, 29.63%). However, it could only maintain 8.05% of the pre-lockdown services (Fig 2, Table 4). With teleconsultation, the optometric (5.54%) and vitreoretinal department (8.28%) were the other least utilized services during the lockdown. Contrarily, the Cornea and Glaucoma departments maintained 30.42% and 29.01% of the pre-lockdown eye care services, respectively (Table 4).

**Table 4:** Percentage decrease in sub-speciality consultation service utility.

Sub-speciality Department	WCR (Pre-Lockdown) WCR _(Pre)_ = 4377	WCR (Post-Lockdown) WCR _(Post)_ = 477	Percentage Decrease in WCR^a^ (%)
Cornea and Refractive Surgery	263	80	69.58
General Ophthalmology	1751	141	91.95
Glaucoma	131	38	70.99
Oculoplastics and Orbital surgery	96	10	89.58
Optometric Services	433	24	94.46
Pediatric Ophthalmology	797	109	86.32
Vitreoretinal Diseases	906	75	91.72

^a^ Percentage of services maintained = 100-% decrease in WCR

### Awareness modes

The automated short message service (SMS) spread the most awareness (52.42%) followed by social media platforms (39.00%). The remaining patients (8.58%) became aware of the teleconsultation programme through the official website as well as their relatives, neighbours and friends.

### Cost-effectiveness

In our study, the weekly cost of the on-site programme was found to be ~56 times higher than that of the teleconsultation programme. The hourly cost of the on-site consultation modality was 2,76,125 PKR (1,649 USD)/week, while it was 4,894 PKR (29 USD)/week for the teleconsultation programme. Moreover, there were no expenses in the teleconsultation programme due to equipment maintenance and repair, hospital building depreciation, equipment depreciation as well as salaries of optometrists and other paramedical staff (Table 5).

**Table 5:** Weekly cost analysis (Hourly basis).

Categories *	Pre-lockdown duration (PKR)	Post lockdown duration (PKR)
Consultancy Charges/Consultant **	5,000	4,688
Internet Charges	1,875	26
Utility Charges	26,250	105
Equipment Maintenance and Repair Charges	15,000	-
Publicity Charges	3,000	75
Hospital Building Depreciation Charges	75,000	-
Equipment Depreciation Charges	112,500	-
Staff Salaries (Optometrists, Nurses, etc.)	37,500	-
Total cost / week ^a^	2,76,125 (1649 USD)	4,894 (29 USD)

* All the costs have been calculated on an hourly basis (48 hours/week)

** 30 and 08 consultants before and during the lockdown, respectively

^a^ Current exchange rate for US dollar (USD) and Pakistani rupee (PKR) is 1 USD = 167. 4 PKR

## Discussion

Our study showed that the teleconsultation programme could maintain 10.89% of the weekly pre-lockdown eye care services. Although the post-lockdown consultation burden was not on par with the pre-lockdown data, eye care services could still be maintained to some extent with teleconsultation during the COVID-19 pandemic. Our pilot study findings also highlight the sociodemographic (female gender, old age, lower-socioeconomic status) and consultation characteristics (new consultations, consultation on weekends) of the patients together with the sub-specialities (optometry, vitreoretinal and oculoplastic services) that could be targeted with focused and inclusive strategies to ensure the equitable utility of teleconsultation services during the pandemic and beyond.

The current study provided evidence that male gender, younger generation and individuals with a higher socioeconomic background were more likely to benefit from teleconsultation. A gender-wise comparison between pre and post-lockdown data demonstrated increased participation of females (~3%) in the teleconsultation programme. This could be attributed to the prevailing conservative cultural traditions in Pakistan that make it difficult for females to travel to healthcare facilities by public transport [46]. The mean age of the patients who made use of teleconsultation was 31.33±19.45 years in comparison to 41.25±23.32 years for the on-site consultation. A plausible explanation could be a more technologically advanced younger generation [47], which is more likely to benefit from technology-driven initiatives. Moreover, more patients from the paying category participated in the teleconsultation programme compared to patients in the “free treatment” category (on-site vs online, Private Category: 0.30% vs 32.21%, Subsidized Payment: 20.19% vs 26.90%, Free Treatment: 75.19% vs 33.12%). Individuals with higher socioeconomic status may have greater access to digital technology and literacy than individuals with a lower socioeconomic status [47]; therefore, they are more likely to adopt and benefit from digital-programmes. On the other hand, patients from the "free treatment" category, in addition to seeking an opinion about their disease, were also interested in obtaining free medicine or surgical treatment that could not be delivered through an online consultation. Further research to explore this disparity must be conducted to ensure equitable eye care.

Overall, the current study demonstrated that more consultations were conducted during weekdays (n = 952, 66.53%) compared to weekends (n = 479, 33.47%) in the lockdown. This is contrary to the trend seen by the Paediatric Ophthalmology department of our hospital, where a significant patient-burden was observed on weekends, as patients’ parents had job obligations and other social duties during the weekdays. Thus, the teleconsultation programme could provide an opportunity for parents to acquire consultation for their children from home, even on the weekdays, without visiting the hospital.

The most common indication for the teleconsultation during the lockdown was red-eye (16.70%, n = 239), which was associated with seasonal ocular allergic disorders (e.g., spring catarrh) and was managed by the corneal service. In addition, trauma and infectious keratitis-related consultations comprised 9.36% (n = 134) and 4.33% (n = 62) of the post-lockdown consultations, respectively. Most of these patients had a history of vegetative trauma that could be linked to the ongoing wheat-harvesting season. Amongst sub-specialities, the teleconsultation programme maintained 29.01% of the pre-lockdown glaucoma department services. While glaucoma is a chronic disease, and the majority of patients require a lifelong therapy for its treatment, these patients found the programme very useful, as they could obtain regular advice regarding issues that occasinally emerge with the use of topical medicines, such as drug allergies or ocular surface problems. They could also inquire about alternatives if some medicine were not available due to the lockdown. However, the requirement of an occasional detailed physical evaluation to monitor the disease progression limits the concept of virtual consultation for the majority of glaucoma patients. Contrarily, optometry along with vitreoretinal and oculoplastics departments’ services were the least utilized during the lockdown (5.54%, 8.28% and 10.42%, respectively). Middle-aged and elderly patients with problems, such as cataract, presbyopia and stable posterior segment pathologies (e.g., stable diabetic retinopathy, stable age-related macular degeneration), likely preferred to wait until routine outdoor services were restored, since their refractive errors and posterior segments could not be evaluated through a teleconsultation. Teleconsultation could be quite a useful way to diagnose and plan the management of many oculoplastics disorders due to their gross appearance [48]; however, these patients were not in a hurry for a consultation during the lockdown, as they had long been aware of their clinical condition. Overall, only 10.76% (n = 154) of patients were referred to the Emergency Department for a detailed assessment. Stable patients were managed through the teleconsultation service hence reducing unnecessary hospital visits. Thus, our programme provided a unique opportunity for telephone-triage of acute problems for referrals, reassurance of stable patients, rescheduling of upcoming appointments, prevention of potential cross-infection and partial maintenance of eye care services during the lockdown in Pakistan.

Some countries such as China are now relaxing the lockdown restrictions since they have attained satisfactory control of SARS-CoV-2 transmission by adopting strict social distancing measures [49]. Such social practices may still be important to avoid another intense pandemic wave [49]. Recently, researchers studied a model of SARS-CoV-2 transmission with time-series data from the USA regarding estimates of immunity, cross-immunity and seasonality for beta corona viruses OC43 and HKU1 [49]. They concluded that recurrent wintertime outbreaks of COVID-19 were likely until 2025. Hence even if the lockdown restrictions were lifted, intermittent or prolonged social distancing might be critically important into 2022 to prevent the resurgence of the contagion and to ensure that critical care capacities are not exceeded [49]. Thus, the expansion of existing teleconsultation services has become imminent [50]. Indeed, once institutions have adopted digital initiatives to maintain clinical services during the pandemic and put the infrastructure in place, the usage of these services will continue.

The surge of digital technology in Pakistan, allowing a wide array of options, may be utilized to promote inexpensive and equitable eye care in remote underprivileged areas [51]. In our study, the weekly cost of the teleconsultation programme was ~56 times lower than that of the on-site programme. However, the comparative cost analysis should be cautiously interpreted because we did not assess the cost of both consultation modalities in relation to the final disease outcome. Compared to teleconsultation, on-site consultation enables clinicians to perform detailed examinations and evaluations through diagnostic tests as well as carry out therapeutic procedures, if necessary, which may improve the final disease outcome. However, more staff members (nurses, optometrists) are required for an efficient on-site consultation programme. Therefore, factors, such as salary bill, utility expenses, and equipment and building depreciation increase the overall operational cost of on-site consultations in comparison to a teleconsultation programme.

While economic broadband services and mobile connectivity would make teleconsultation affordable and accessible, targeted-marketing strategies (e.g., SMS to female and elderly patients) may also be adopted to expand the outreach and scope of digital initiatives. Using teleconsultation, “Home Clinics” could be arranged for females and elderly patients who might find it challenging to come to healthcare premises due to various social issues and cultural sensitivities [52, 53]. Mobile phones and Internet-based telecommunication tools could also be used for remote eye care management and bedside monitoring with video assessment and patient-doctor interaction [54]. Moreover, patients, especially females and the elderly, could be trained on available self-testing mobile-applications that could be incorporated into the teleconsultation programmes to offer general and specialized eye care (e.g., optometry and vitreoretinal services, visual field testing) [55–66]. With teleconsultation, cybersecurity and limited online payment platforms may be concerning; hence, efforts are needed to address these issues. Future work by our group is underway to study a scoring system to help clinicians register patients in a teleconsultation programme.

In this study, a detailed assessment of the sub-speciality burden in ophthalmology was conducted, which determined the utilization trends of different sub-specialities and identified factors that may hamper eye care equity and access during the COVID-19 pandemic. A multivariate binary logistic regression analysis was performed, but it was not included in the final results because of minimal predictive power and a very limited set of available possible predictors. Moreover, patients’ feedback regarding the teleconsultation programme that could have better described the utility of this service could not be collected. Future work studying this aspect is underway.

In conclusion, teleconsultation utility in ophthalmology is a relatively unexplored frontier, especially in developing countries such as Pakistan. The current study showed that teleconsultation could partially maintain eye care services during a lockdown. It also identified the key sociodemographic characteristics of the patients along with the sub-specialities that could be targeted with policy-based interventions to advance equitable eye care and promote teleconsultation utilization. In the words of sages, "a crisis provides an opportunity"; the pandemic too provides an opportunity for the further fusion of the global health sector with optimal digital technology. Effective and immediate utilization of digital initiatives to combat this colossal global health challenge would, in all likelihood, increase the general acceptance of such technologies for other sub-specialities of healthcare in the future.

## Supporting information

S1 Appendix Data variables of the study. (SAV)
